# Syndecan-1 and Free Indoxyl Sulfate Levels Are Associated with miR-126 in Chronic Kidney Disease

**DOI:** 10.3390/ijms221910549

**Published:** 2021-09-29

**Authors:** Ophélie Fourdinier, Griet Glorieux, Benjamin Brigant, Momar Diouf, Anneleen Pletinck, Raymond Vanholder, Gabriel Choukroun, Francis Verbeke, Ziad A. Massy, Valérie Metzinger-Le Meuth, Laurent Metzinger, on behalf of the European Uremic Toxin Work Group-EUTox

**Affiliations:** 1 Nephrology Dialysis and Transplantation Department, Amiens University Hospital, 80054 Amiens, France; fourdinier.ophelie@chu-amiens.fr (O.F.); Choukroun.Gabriel@chu-amiens.fr (G.C.); 2 Nephrology Section, Department of Internal Medicine and Pediatrics, Ghent University Hospital, Corneel Heymanslaan 10, 9000 Ghent, Belgium; Griet.Glorieux@UGent.be (G.G.); Anneleen_Pletinck@hotmail.com (A.P.); Raymond.Vanholder@UGent.be (R.V.); Francis.Verbeke@UZGent.be (F.V.); 3 UPJV HEMATIM UR 4666, C.U.R.S, Université de Picardie Jules Verne, CEDEX 1, 80025 Amiens, France; benjamin.brigant@gmail.com (B.B.); valerie.metzinger@univ-paris13.fr (V.M.-L.M.); 4 Biostatistics Unit, Clinical Research and Innovation Department, Amiens-Picardie University Hospital, 80054 Amiens, France; Diouf.Momar@chu-amiens.fr; 5 Ambroise Paré Hospital, Division of Nephrology, APHP, Paris Ile de France Ouest (UVSQ) University, et INSERM 1018 Eq. 5, CESP, Boulogne Billancourt et Villejuif, 92100 Paris, France; ziad.massy@apr.aphp.fr; 6 INSERM UMRS 1148, Laboratory for Vascular Translational Science (LVTS), UFR SMBH, Université Sorbonne Paris Nord, CEDEX, 93017 Bobigny, France

**Keywords:** uremic toxins, indoxyl sulfate, microRNA, miR-126, chronic kidney disease, biomarker, syndecan-1, endothelium dysfunction

## Abstract

Chronic kidney disease (CKD) is a major cause of death worldwide and is associated with a high risk for cardiovascular and all-cause mortality. In CKD, endothelial dysfunction occurs and uremic toxins accumulate in the blood. miR-126 is a regulator of endothelial dysfunction and its blood level is decreased in CKD patients. In order to obtain a better understanding of the physiopathology of the disease, we correlated the levels of miR-126 with several markers of endothelial dysfunction, as well as the representative uremic toxins, in a large cohort of CKD patients at all stages of the disease. Using a univariate analysis, we found a correlation between eGFR and most markers of endothelial dysfunction markers evaluated in this study. An association of miR-126 with all the evaluated uremic toxins was also found, while uremic toxins were not associated with the internal control, specifically *cel-miR-39*. The correlation between the expression of endothelial dysfunction biomarker Syndecan-1, free indoxyl sulfate, and total p-cresyl glucuronide on one side, and miR-126 on the other side was confirmed using multivariate analysis. As CKD is associated with reduced endothelial glycocalyx (eGC), our results justify further evaluation of the role of correlated parameters in the pathophysiology of CKD.

## 1. Introduction

Chronic kidney disease (CKD) is a leading cause of death around the world and is associated with a major risk for cardiovascular (CV) and all-cause mortality [1,2]. CKD is divided into five stages with increasing severity and patients with stage 5 develop kidney failure. As the disease progresses, the kidney becomes less efficient, resulting in the accumulation of a number of uremic toxins in the blood [1]. To monitor CKD progression, the criteria used by clinicians are the doubling in serum creatinine, the initiation of kidney replacement therapy, or reduction in the estimated glomerular filtration rate (eGFR) by 15 mL/min/1.73 m^2^ or to 10 mL/min/1.73 m^2^. Oftentimes, the most common indicator of kidney function, namely eGFR, is used. However, eGFR has major limitations such as a weak sensitivity in detecting early stage CKD and poor prediction of the course of CKD [3]. It is thus necessary to promote the discovery and validation of innovative biomarkers to improve CKD research and treatment. miRNAs are key controllers of the gene program and thus are potential choices to modulate this process [4]. They have already been abundantly studied in the nephrology field (reviewed in [5]) but their use as biomarkers needs to be validated in large cohorts. We have previously shown that the serum levels of two miRNAs, namely miR-126 and miR-223, is decreased in a large cohort of CKD patients [6]. Serum levels of both miRNAs was associated with all-cause mortality, but also with CV and renal events. A failure in the regulatory role of mR-126 in endothelial function during CKD has thus been suggested [7,8,9,10,11]. To better understand the role of this non-coding RNA in progressing stages of CKD, we aimed in this work to correlate its expression with markers of endothelial dysfunction, on one hand, and uremic toxins, on the other hand.

Endothelial dysfunction is a pathological condition of the vascular endothelium that is a mainstay of CKD and is known to increase the risk for CV disease [12]. There are several recently discovered markers of endothelial damage that can be used to assess this process in CKD [13]. Among these, syndecan-1 (Sdc-1) is part of a transmembrane heparan sulfate glycoprotein family, known to be sponges for growth factors and chemokines which are involved in various cellular processes [14]. The vascular intercellular adhesion molecule (VCAM)-1 is a heavy glycosylated cell surface protein, typically expressed on endothelial cells. VCAM-1 facilitates kidney macrophage recruitment during vessel damage [15]. Matrix metalloproteinase-7 (MMP-7) is involved in extracellular matrix breakdown during tissue remodeling [16]. MMP-7 is upregulated in CKD and was thus suggested as a biomarker of endothelial dysfunction in renal disorders [17]. Angiopoietin-2 (ANGPT2) alters cell–matrix contacts, thus impacting endothelial cell apoptosis, migration, and proliferation [18].

Another feature of CKD is the accumulation of uremic toxins in the blood [19]. They are subdivided in (a) small, water-soluble compounds (e.g., inorganic phosphate); (b) middle molecules; and (c) protein-bound compounds (e.g., indoxyl sulfate (IS)) based on their molecular weight and protein-binding capacity, which reflects their behavior during dialysis. Uremic toxins are known to affect vascular physiology by impacting endothelial, platelet, and immune dysfunction [13,19]. One of the challenges concerns understanding how uremic toxins can affect gene regulation to trigger CKD and conversely how one can use this knowledge to slow down CKD complications and/or progression.

The present study is a follow-up of our previous work [6]. The characteristics of the cohort were previously described in detail in [6,13]. This large cohort was divided into seven CKD groups (stages G1,G2, G3a, G3b, G4, G5, and G5D for patients on dialysis) and one healthy group [6,13]. In this cohort, the all-cause mortality as well as the CV and renal events are archived as endpoints over a 5.5 year period. It has been shown that miR-126 levels were significantly lower from CKD stage G2 onwards compared to the controls [6]. When considering overall mortality, patients with levels of miR-126 below the median had a lower survival rate. Similar results were observed for CV and renal events [6]. In the present study, we tried to detect eventual associations between miR-126 expression and markers of endothelial dysfunction, on one hand, and uremic toxins, on the other hand, to obtain a better grasp of the interactions of these molecules in the CKD.

## 2. Results

### 2.1. miR-126 Serum Levels Are Associated with Syndecan-1

**Figure 1:**
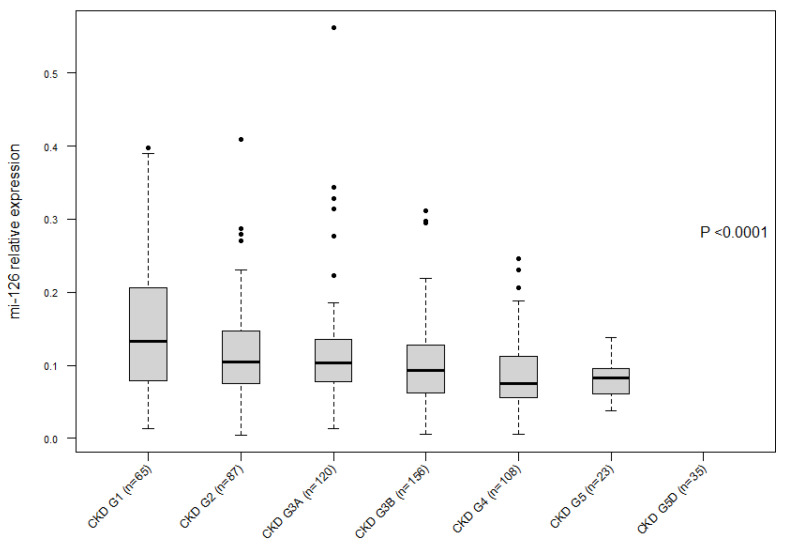
Seric expression of miR-126 decreases according to the CKD patient’s stage.

**Figure 2:**
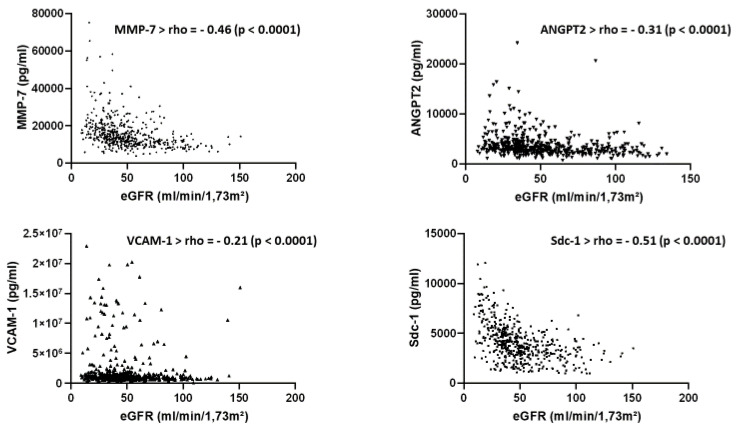
Association of the estimated glomerular filtration rate (eGFR) with the endothelial markers matrix metalloproteinase-7 (MMP-7); Angiopoietin 2 (ANGPT2); vascular cell adhesion protein 1; (VCAM-1) and Syndecan-1 (Sdc-1) in patients in different stages of CKD (Spearman’s rank correlation coefficient).

The baseline characteristics of the cohort analyzed in the present study are presented in Supplementary Table S1. Figure 1 confirms a decrease in the seric expression of miR-126 according to the CKD stage, as already described in Fourdinier et al. [6] using the same cohort with slightly different demographics. Figure 2 shows a clear correlation between eGFR and the four endothelial dysfunction markers evaluated in this study, i.e., Sdc-1, MMP-7, ANGPT2, and VCAM-1, confirming their value as biomarkers in this pathology.

The association of miR-126 with the endothelial markers MMP-7, ANGPT2, Sdc-1, and VCAM-1 was studied by using a Spearman’s correlation. miR-126 was found to be associated with eGFR, Sdc-1, VCAM-1, and MMP-7 but not with ANGPT2 (Table 1).

Next, a multiple linear regression analysis of the three biomarkers significantly associated with circulating levels of miR-126 was performed (Table 2). We confirmed an already found independent link between miR-126 and the platelet count, hemoglobin, and age [6]. Interestingly, miR-126 was shown to be independently correlated with Sdc-1 expression, which was not the case for MMP-7 and VCAM-1.

### 2.2. miR-126 Serum Levels Are Associated with Free IS and Total pCG

**Figure 3:**
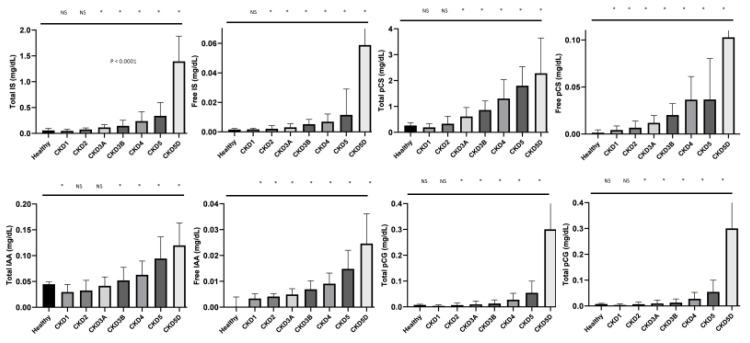
Levels of uremic toxins over the different stages of CKD. * *p* < 0.05 vs healthy. Abbreviations: NS, non-significant; IS, indoxyl sulfate; pCS, para-cresyl-sulfate; pCG, p-cresyl glucuronide; and IAA, indole acetic acid.

With the intent to detect links between circulating levels of miR-126 and levels of uremic toxins, a set of correlation analyses between four of the main uremic toxins, IS, PCS, PCG, and IAA was performed. Both total and free plasma concentrations were considered. Figure 3 shows that all the uremic toxin levels increase with progressive stages of CKD, as was already published for the complete cohort (Figure 3) [13].

An association of miR-126, using the Spearman’s rank correlation coefficient, was found for all the evaluated uremic toxins, while uremic toxins were not associated with the internal control, namely *cel-miR-39* (Table 3). However, in a multiple linear regression analysis of the variables, circulating levels of miR-126 were no longer independently associated with most uremic toxin levels, except for free IS and total pCG (Table 4). An independent association of miR-126 with age, hemoglobin and platelet count, as previously described, was confirmed [6].

Finally, we looked at the interactive predictive role of miR-126, SDC-1, and free IS on overall survival and CV event-free survival. We determined the Integrated Discrimination Index (IDI), the Net Reclassification Improvement (NRI), Harrell’s C-index, and the Akaike Information Criterion (AIC) using our parameters (Supplementary Table S2). Out of the three parameters, free IS was the only one that was significantly altered regarding the hazard ratio. Accordingly, this parameter improved IDI (at 36 months, concerning overall survival, and at 13 and 36 months, concerning CV event-free survival).

## 3. Discussion

Our former study was the first to evaluate the serum expression of miR-126 as a potential diagnostic and prognostic biomarker in a large cohort of patients with CKD at various stages of the disease, including a group of controls without CKD [6]. In this work, we had access to a wide range of laboratory parameters with a long-term follow-up, enabling the estimation of serum miRNA levels on hard outcomes. We showed that patients having levels of miR-126 below the median had a lower survival rate with a parallel increase in CV and kidney events. The link between the serum levels of miRNA and mortality, cardiovascular events, or kidney events in CKD was, however, dependent on eGFR [6]. This information did not, however, exclude a potential role of miR-126 in the pathophysiology of CKD, thus we wanted to further explore this hypothesis in the present follow-up study. In support of this, Scullion et al. recently showed that miR-126 serum was reduced in rats with nephrotoxic nephritis and in humans with both acute endothelial and renal injury compared to healthy controls [11]. Furthermore, in this work, miR-126 increased dramatically post-treatment but remained lower than in healthy animals.

In a previous work focusing on the present cohort (limited by the population, in comparison to that studied here, at 523 patients with only non-dialysis CKD stage G1-G5) [13] free pCS showed the highest association with cardiovascular outcomes in non-dialyzed patients with CKD. In the present work, studying the same cohort, we correlated the expression of several endothelial dysfunction biomarkers with miR-126 serum levels. We found that Sdc-1, on one side, and miR-126, on the other side, were correlated in a multivariate analysis. CKD was associated with reduced endothelial glycocalyx (eGC), a combination of anionic polymers on the luminal surface of endothelial cells, which protects the vessel wall against injuries that would result in endothelial dysfunction. It was shown that shedding of Sdc-1 from eGC is increased in CKD patients [20]. Interestingly, this is most prominent in patients receiving dialysis, suggesting that this association is CKD-stage dependent [21]. Upregulation of Sdc-1 mRNA indicates the onset of an acute injury superimposed on chronic kidney disease [22]. Additionally, we have described a clear link between miR-126 and vascular injury in CKD models [7,8,23], as well as a decrease in the serum levels of this miRNA in CKD patients [6]. In addition, Scullion et al. found a strong correlation between (1) circulating miR-126 and (2) pulse-wave velocity and Asymmetric Dimethylarginine (ADMA), both markers of endothelial function [11]. Our novel results strengthen the hypothesis of a link between miR-126 levels and endothelial dysfunction in a large cohort of CKD patients. Moreover, miR-126 was shown to induce CXCL12-induced angiogenesis via its target gene Spred-1, showing that its expression could be involved in vascularization and tissue regeneration [24]. In HUVEC endothelial cells, angiogenesis and migration induced by CXCL12 were abolished after miR-126 inhibition. Finally, a link between miR-126 cellular levels and Sdc-1 was already found in prostate cancer cells, where Sdc-1 silencing led to decreased expression of miR-126 [25]. In this model, miR-126 inhibited cell growth and induced senescence.

The detrimental effect of IS and other protein-bound toxins has been amply demonstrated in the CKD course [19,26]. IS is involved in the pathophysiology of cardiovascular complications in CKD [19,26]. In this regard, it is interesting that miR-126 is associated with free IS levels in a multivariate analysis. It was published that a higher free level of IS was associated with a higher relative risk of mortality compared to total levels, which is in accordance with our results in which free IS levels were associated with miR-126 and not total IS [27]. When we studied the interactive predictive role of free IS, we found that free IS improved IDI, showing an additive predictive role of this potential new biomarker on hard outcomes in CKD, beyond the traditional prognostic markers such as baseline eGFR. However, we also found an association of total pCG levels and not pCG-free levels with miR-126. We did not find any association with free nor total pCS. Conjugation of p-cresol by the microbiome predominantly creates pCS and, at a lesser degree, pCG [28,29]. As already stated in a previous study on this cohort [13], free pCS showed the highest association with cardiovascular outcomes in non-dialyzed patients with CKD. IS acts more on vascular remodeling, whereas pCS reduces endothelial cells’ viability, as reviewed recently in [30]. Moreover, Carmona et al. published that IS activates the production of microvesicles in endothelial cells and suggested that the miRNA contents in these vesicles could be altered [31]. The same group showed that microvesicles generated in endothelial cells treated with pCS also produce microvesicles that interfere with the endothelial repair and increase the senescence of mature endothelial cells [32]. One can suggest that the two uremic toxins trigger the formation of distinct vesicles and that each category will have different biological roles. This would be consistent with our observations that only IS, and not pCS, is correlated with miR-126. This seems to indicate that the link between miR-126 and the two toxins derived from p-cresol is not identical when considering hard outcomes. In contrast to pCS, p-CG fails to promote insulin resistance at least in rodents [33]. This discrepancy could explain why we did not find equivalent results for pCS and pCG in regard to miR-126 association.

In summary, our results suggest a role of miR-126 in endothelial dysfunction in a large cohort of CKD patients. Other miRNAs have been described as potential biomarkers of CKD and/or cardiovascular damage [5], such as miR-133a [33]. In small cohorts, miR-133 and miR-451 were described as early predictors of diabetic nephropathy [34,35]. miR-125b and miR-155 have been implicated in CKD progression within both in vivo and in vitro models [36,37]. miR-93 and miR-223 are also among the candidates that were shown as associated to the CKD process in medium and large cohorts [6,38]. This is, however, the first time that an association between a miRNA, an endothelial dysfunction marker, and several uremic toxins has been shown in a large CKD patient cohort.

## 4. Material and Methods

### 4.1. The Study Population

In total, 562 patients afflicted with CKD stages G1 to G5 (Kidney Disease Outcomes Quality Initiative (KDOQI)) were included in this single-center study at the outpatient clinic of the Nephrology Section of the Ghent University Hospital (Ghent, Belgium). Inclusion into the study was from January 2011 to January 2014. The exclusion criteria were an age under 18, active infection, pregnancy, malignancy, or transplantation history. Outcome parameters were monitored for 5,5 years until June 2017. Patients with CKD were categorized into subgroups according to their estimated glomerular filtration rate (eGFR) using the Chronic Kidney Disease Epidemiology Collaboration (CKD-EPI), following KDOQI guidelines. Eight subgroups were compared, including six CKD subgroups not on dialysis (eGFR: over 90, 60–89, 45–59, 30–44, and 15–29, and below 15 mL/min/1.73 m²), one CKD subgroup on RRT (stage G5D), and healthy controls. Hypertension was defined as a systolic blood pressure >140 mmHg and/or a diastolic blood pressure >90 mmHg, or as the need for anti-hypertensive medication. In this study, we mostly studied the six CKD subgroups not on dialysis. To address the batch effect during the determination of the serum miR-126 levels and other endothelial markers, samples were collected, aliquoted, and stored at −80 °C until batch analysis. A new aliquot was used for each analysis and no thaw/freeze cycle was applied. For the endothelial markers, a human magnetic luminex assay was used and a premix multiplex was ordered. The same lot number (L123484) was used for all patient samples.

### 4.2. Ethical Approval

This study was approved by the local ethical committee at the Ghent University Hospital (2010/033; B67020107926). Written informed consent was obtained from all participants. The study complied with the tenets of the Declaration of Helsinki and its amendments.

### 4.3. RNA Extraction and Quantification of Serum miRNA Levels

RNA was extracted from patient blood and RT-qPCR was performed as described in detail previously [6]. Venous blood samples were extracted in Venosafe serum (Terumo Europe, Leuven, Belgium) tubes, left at room temperature for 30 min, aliquoted at 4 °C, and stored at −80 °C until analysis. Total RNA was extracted using the miRNeasy Serum/Plasma kit (Qiagen, Germany) according to manufacturer’s instructions. A given quantity of exogenous *Caenorhabditis elegans* miR-39 was spiked-in to be used as an internal control, as described in [6]. RNA was kept at −80 °C until further use. NanoDrop spectrophotometer analysis was used to assess the RNA quality (Thermo Fisher Scientific, Waltham, MA, USA). RNA was reverse-transcribed into cDNA with the help of TaqMan miRNA-specific primers and the TaqMan microRNA reverse transcription kit (Applied Biosystems, Waltham, MA, USA). The relative expression level of miR-126 was estimated for each patient using the 2-ΔCq method (ΔCq  =  Cq (miRNA) − Cq (cel-miR-39)). Only one person (O.F.), blinded for the patients, performed all experiments to avoid bias.

### 4.4. Endothelial Dysfunction Markers Quantification

These quantifications were described in detail in [13]. Briefly, Magnetic Luminex Assays (R&D Systems, Minneapolis, MN, USA) were used to simultaneously measure the levels of the endothelial dysfunction markers among which matrix metalloproteinase (MMP-7), vascular cell adhesion molecule-1 (VCAM-1), angiopoietin-2 (ANGPT2), and syndecan-1 (Sdc-1) were present in plasma samples as previously described. The category number of the magnetic luminex assay premix multiplex was LXSAHM. Experimental data was analyzed by fitting a 5-parameter logistic curve to the standard analyte curves. The lowest standard of the calibration curves were 115,08 pg/mL for SDC-1; 8264,8 pg/mL for VCAM-1; 91,3 pg/mL for ANGPT-2; and 320.61 pg/mL for MMP-7.

### 4.5. Uremic Toxins Quantification

These quantifications were described in detail in [13]. Briefly, concentrations of the total and free concentrations of the uremic toxins indoxyl sulfate (IS), para-cresyl-sulfate (pCS), p-cresyl glucuronide (pCG), and indole acetic acid (IAA) were determined using ultrahigh-performance liquid chromatography (UPLC) with ultraviolet and fluorescence detection.

### 4.6. Statistical Analyses

Correlations between patient characteristics and various levels were performed with Spearman’s rank correlation tests. Multiple linear regression analyses were performed to investigate the association between the parameters and the expression of miRNAs. In the case of non-normal distribution, the data were log-transformed. All the parameters with a *p*-value of <0.05 in the univariate analysis were analyzed by multivariate analysis. Statistical analyses were conducted using GraphPad Prism^®®^ software version 6 and SPSS^®®^ software version 21 unless stated otherwise. The Harrell’s C-index and AIC were estimated using the Cox model, whereas NRI and IDI were calculated using the package « SurvIDINRI » of software R version 4.0.5 through interface RStudio software version 1.0.143—© 2009–2016 RStudio, Inc. The threshold for statistical significance was defined as *p* < 0.05 in all tests.

## 5. Conclusions

miRNAs are promising new biomarkers in the CKD field. They need, however, more validation. Here, we show in a large cohort that the alterations in miR-126 levels are correlated with markers of endothelial dysfunction and cardiovascular disease, further expanding knowledge on the physiopathology of this non-coding RNA in CKD. In the future, long non-coding RNAs can also be developed as better biomarkers of CKD, as they are known to be more tissue-specific than miRNAs [39].

## Figures and Tables

**Table 1:** Association of miR-126 with endothelial markers using univariate analysis.

	miR-126	
Parameters	ρ	*p*
eGFR (*n* = 562)	−0.23	***p* < 0.0001**
Age	−0.23	***p* < 0.0001**
Platelet count	0.18	***p* < 0.0001**
Hemoglobin	0.24	***p* < 0.0001**
MMP-7 (*n* = 515)	−0.23	***p* < 0.0001**
Sdc-1 (*n* = 515)	−0.29	***p* < 0.0001**
VCAM-1 (*n* = 513)	−0.09	***p* = 0.03**
ANGPT-2 (*n* = 515)	−0.05	*p* = 0.25

Angiopoietin 2 (ANGPT2); matrix metalloproteinase-7 (MMP-7); Syndecan-1 (Sdc-1); and vascular cell adhesion protein 1 (VCAM-1). Spearman’s rank correlation coefficient is indicated by ρ. Significant correlations are marked in bold.

**Table 2:** Multiple linear regression analysis of endothelial markers independently associated with circulating levels of miR-126.

miR-126	β	*p*-Value
eGFR (CKD-Epi) (per 1 mL/min/1.73 m²)	0.08	0.17
Platelet count (per 1/mm^3^)	0.12	**0.005**
Hemoglobin (per 1 g/dL)	0.10	**0.03**
Age (per year)	−0.18	**0.001**
MMP-7 (mg/dL)	−0.07	0.17
Sdc-1 (mg/dL)	−0.12	**0.01**
VCAM-1 (mg/dL)	0.02	0.71

Abbreviations: MMP-7, matrix metalloproteinase-7; Sdc-1, Syndecan-1; and VCAM-1, vascular cell adhesion protein 1. Spearman’s rank correlation coefficient is indicated by ρ. Significant correlations are marked in bold.

**Table 3:** Univariate association of miR-126 and free and total blood levels of uremic toxins (Spearman’s rank correlation coefficient is indicated by ρ).

	miR-126		Cel-miR-39	
Parameters	ρ	*p*	ρ	*p*
Total IS (*n* = 625)	−0.30	***p* < 0.0001**	−0.02	NS
Free IS (*n* = 625)	−0.33	***p* < 0.0001**	−0.02	NS
Total pCS (*n* = 625)	−0.27	***p* < 0.0001**	−0.01	NS
Free pCS (*n* = 625)	−0.29	***p* < 0.0001**	−0.07	NS
Total pCG (*n* = 625)	−0.27	***p* < 0.0001**	−0.02	NS
Free pCG (*n* = 625)	−0.26	***p* < 0.0001**	−0.02	NS
Total IAA (*n* = 625)	−0.19	***p* < 0.0001**	0.008	NS
Free IAA (*n* = 625)	−0.25	***p* < 0.0001**	−0.06	NS

Abbreviations: IS, indoxyl sulfate; pCS, para-cresyl-sulfate; pCG, p-cresyl glucuronide; and IAA, indole acetic acid. Significant correlations are marked in bold.

**Table 4:** Multiple linear regression analysis of uremic toxins independently associated with circulating levels of miR-126.

miR-126	β	*p*-Value
eGFR (CKD-Epi) (per 1 mL/min/1.73 m²)	0.13	**0.03**
Platelet count (per 1/mm^3^)	0.15	**0.001**
Hemoglobin (per 1 g/dL)	0.13	**0.003**
Age (per year)	−0.13	**0.01**
Sdc-1 (mg/dL)	−0.12	**0.01**
Total IS (mg/dL)	0.07	0.37
Free IS	−0.26	**0.03**
Total pCS (mg/dL)	−0.06	0.35
Free pCS	0.04	0.64
Total pCG (mg/dL)	−0.54	**0.02**
Free pCG	0.58	0.11
Total IAA (mg/dL)	−0.10	0.34
Free IAA	0.13	0.24

Abbreviations: IS, indoxyl sulfate; pCS, para-cresyl-sulfate; pCG, p-cresylglucuronide; and IAA, indole acetic acid. Significant correlations are marked in bold.

## Supplementary Materials

The following are available online at https://www.mdpi.com/article/10.3390/ijms221910549/s1.
