# Supplementary material for: Syndecan-1 and Free Indoxyl Sulfate Levels Are Associated with miR-126 in Chronic Kidney Disease
Source: Int J Mol Sci. 2021 Sep 29;22(19):10549. doi: 10.3390/ijms221910549 (PMC8508835; doi:10.3390/ijms221910549)
Supplement: Supplementary file 1 [file ijms-22-10549-s001.zip › Supplementary Table 2 Fourdinier et al.pdf]

Supplementary Table 2. Interactive predictive role of free IS, SDC-1 and miR-126 on hard outcomes in CKD.

A. Overall Survival	Model0 : CKD-Epi	Model1 : CKD-Epi miR-126	Model2 : CKD-Epi Sdc-1	Model3 : CKD-Epi **Free IS**
HR (95%)	0.96[0.95 ; 0.97]	0.96[0.95 ; 0.97] 1.67[0.08 ; 35.45]	0.97[0.96 ; 0.98] 1.08[0.98 ; 1.19]*	0.97[0.96 ; 0.98] **1.11[1.03 ; 1.19]****
P-value	<0.0001	<0.0001 0.742	<0.0001 0.111	<0.0001 **0.0055**
12month-IDI		-0.03% [-0.30%; 0.23%]	0.82% [-0.09%; 4.52%]	0.43% [-0.03%; 1.19%]
12month-½NRI		-12.3% [-28.3%; 29.6%]	13.9% [-19.4%; 34.8%]	9.7% [-18.2%; 43.0%]
36month-IDI		0.009% [-0.12%; 0.82%]	1.0% [-0.2%; 4.5%]	**1.43% [0.17%; 3.34%]**
36month- ½NRI		0.83% [-9.8%; 12.0%]	1.6% [-17.2%; 14.8%]	0.3% [-17.1%; 16.1%]
Harrell's c-index	0.714±0.02	0.713±0.02	0.717±0.02	0.718±0.02
AIC	1606	1607	1605	1603
B. CV Event-free Survival	Model0 : CKD-Epi	Model1 : CKD-Epi miR-126	Model2 : CKD-Epi Sdc-1	Model3 : CKD-Epi **Free IS**
HR (95%)	0.96[0.95 ; 0.97]	0.96[0.95 ; 0.97] 1.54[0.07 ; 33.11]	0.97[0.96 ; 0.98] 1.07[0.97 ; 1.18]*	0.96[0.95 ; 0.97] **1.11[1.03 ; 1.19]****
P-value	<0.0001	<0.0001 0.781	<0.0001 0.157	<0.0001 **0.0055**
12month-IDI		-0.03% [-0.24%; 0.27%]	0.72% [-0.19%; 4.22%]	**0.43% [0.00%; 1.69%]**
12month-½NRI		-12.3% [-29.0%; 30.6%]	13.9% [-25.8%; 36.4%]	9.86% [-13.9%; 37.7%]
36month-IDI		-0.01% [-0.15%; 0.70%]	0.88% [-0.32%; 4.55%]	**1.41% [0.23%; 3.34%]**
36month- ½NRI		-0.17% [-9.8%; 16.0%]	2.07% [-23.50%; 14.24%]	-0.12% [-16.7%; 17.5%]
Harrell's c-index	0.714±0.02	0.713±0.02	0.717±0.02	0.718±0.02
AIC	1606	1607	1605	1603

* relative decrease associated with an increase of 1000 units of variable Sdc1-
** relative decrease associated with an increase of 0.01 unités of variable IS_free
½NRI is the category-free NRI (net reclassification improvement) de Pencina et al (DOI: 10.1002/sim.4085)
HR=hazard ratio
IDI=Integrated Discrimination Index
AIC=Akaike Information Criterion
